# Mortality Trends Among Early Adults in the United States, 1999-2023

**DOI:** 10.1001/jamanetworkopen.2024.57538

**Published:** 2025-01-31

**Authors:** Elizabeth Wrigley-Field, Rafeya V. Raquib, Kaitlyn M. Berry, Keeley J. Morris, Andrew C. Stokes

**Affiliations:** 1 Department of Sociology, University of Minnesota, Minneapolis; 2 Minnesota Population Center, University of Minnesota, Minneapolis; 3 Department of Global Health, Boston University School of Public Health, Boston, Massachusetts; 4 Division of Epidemiology and Community Health, University of Minnesota School of Public Health, Minneapolis

## Abstract

This cross-sectional study examines trends in mortality rates among adults aged 25 to 44 years across the pre–COVID-19 pandemic, pandemic, and postpandemic periods.

## Introduction

Mortality rate improvements have stalled or reversed for many US population groups since approximately 2010. Although these trends have been described,^1,2,3,4,5^ few studies have focused on early adulthood (ages 25-44 years) specifically—the period during which many health behaviors are established. A 2021 report documented increasing mortality at these ages across many causes of death from 2010 to 2017.^6^ The current study extends prior work by documenting trends in early adult mortality across the pre–COVID-19 pandemic, pandemic, and postpandemic periods.

## Methods

This cross-sectional study was deemed exempt from review and informed consent by the University of Minnesota institutional review board because the data did not include human participants. We followed the Strengthening the Reporting of Observational Studies in Epidemiology (STROBE) reporting guideline.

We calculated monthly mortality rates using cause-specific death counts from Centers for Disease Control and Prevention WONDER queries (eMethods in Supplement 1) and midyear population estimates from the US Census Bureau for the adults aged 25 to 44 years between 1999 and 2023. Mutually exclusive and exhaustive underlying cause-of-death categories were adapted from prior work (eMethods in Supplement 1).^6^ Autoregressive integrated moving average models were estimated for each cause, using a baseline of 1999 to 2010, to project expected mortality trends for 2011 to 2023.^3^ Cause-specific excess mortality was calculated as the difference between observed and expected mortality for each year. We estimated CIs estimated using bootstrapping. Further methodological details are provided in the eMethods in Supplement 1.

## Results

We analyzed 3 392 364 deaths among the full US population aged 25-44 years from 1999 to 2023. Mortality increases across most causes of death produced substantial excess deaths compared with extrapolations of pre-2011 trends (Figure). Early adult excess mortality was 34.6% higher than expected in 2019 and then further accelerated during the COVID-19 pandemic. In 2021, all-cause excess mortality was nearly 3 times what it had been in 2019 (116.2 vs 41.7 deaths per 100 000 population). In 2023, excess mortality decreased, but only to approximately midway between its 2019 and 2021 levels (79.1 deaths per 100 000 population). As a result, early adult mortality was 70.0% higher in 2023 than it would have been had pre-2011 trends continued, reflecting 71 124 excess deaths (Table).

**Figure.**
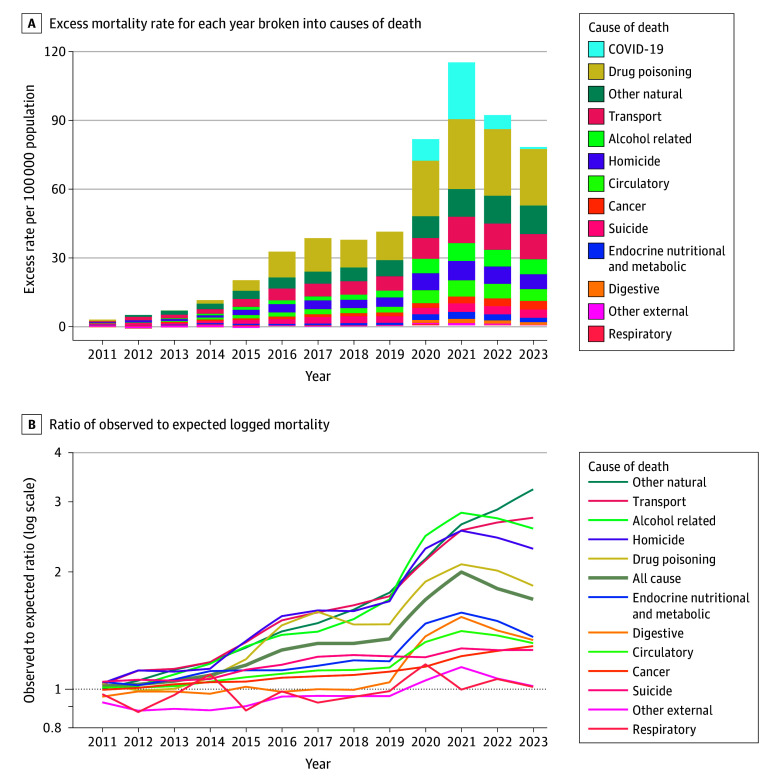
Excess Mortality Rates by Cause Among Adults Aged 25-44 Years, 2011-2023 Excess mortality rates are estimated as the difference between the mortality rate and the rate projected from the baseline (1999-2010) trend, measured in deaths per 100 000 population. A, COVID-19 is given as an underlying cause of death for the purpose of contextualizing the other causes; given that COVID-19 was a new cause of death in 2020, we present all COVID-19 deaths as excess mortality to emphasize the change from the prepandemic period and facilitate comparison to changes in other causes of death. B, COVID-19 is omitted since there was no baseline trend and a line for all-cause mortality is added. Causes of death are listed in highest to lowest order in 2023.

**Table.** Observed, Counterfactual, and Excess Deaths, 2023

Cause of death	Observed deaths, No.	Counterfactual deaths, No. (95% CI)	Ratio of observed to counterfactual	Excess deaths	
				No. (95% CI)	% Of total
All-cause^a^	172 785	101 661 (67 231 to 135 569)	1.70	71 124 (37 216 to 105 554)	NA
Drug poisoning	48 884	26 566 (10 076 to 42 816)	1.84	22 318 (6068 to 38 808)	31.8
Other natural	16 265	5007 (−11 511 to 21 262)	3.25	11 258 (−4997 to 27 776)	16.0
Transport	15 551	5659 (−4120 to 15 284)	2.75	9892 (267 to 19 671)	14.1
Alcohol related	9763	3786 (2101 to 5469)	2.58	5977 (4294 to 7662)	8.5
Homicide	10 257	4481 (−1938 to 10 821)	2.29	5776 (−564 to 12 195)	8.2
Circulatory	19 701	15 015 (8190 to 21 736)	1.31	4686 (−2035 to 11 511)	6.7
Cancer	15 137	11 738 (7713 to 15 770)	1.29	3399 (−633 to 7424)	4.8
Suicide	15 658	12 421 (11 084 to 13 759)	1.26	3237 (1899 to 4574)	4.6
Endocrine nutritional and metabolic	6536	4803 (2622 to 6957)	1.36	1733 (−421 to 3914)	2.5
Digestive	4278	3201 (1535 to 4874)	1.34	1077 (−596 to 2743)	1.5
COVID-19	766	NA	NA	766 (NA)	1.1
Other external	6194	6082 (928 to 11 258)	1.02	112 (−5064 to 5266)	0.2
Respiratory	3795	3743 (2146 to 5349)	1.01	52 (−1554 to 1649)	0.1

Abbreviation: NA, not applicable.

^a^ All-cause excess mortality was calculated directly based on autoregressive integrated moving average models estimated on the historical all-cause mortality series. For comparison, excess all-cause mortality would be marginally lower (decomposition discrepancy of 841) if it were obtained from the sum of estimated excess deaths across each cause of death. The total number of excess deaths used to calculate the percentage of total excludes the decomposition discrepancy.

The 5 causes of death that collectively accounted for almost three-quarters of the early adult excess mortality in 2023 were drug poisoning (31.8% of excess mortality), the residual natural-cause category (16.0%), transport-related deaths (14.1%), alcohol-related deaths (8.5%), and homicide (8.2%). Additionally, the combined contribution of cardiometabolic conditions, including circulatory and endocrine, metabolic, and nutritional, was substantial (9.2%).

## Discussion

This cross-sectional study found that compared with trends from the early 2000s, early adult mortality in the US has risen substantially in 2 stages: 2011 to 2019 and 2020 to 2023. Although mortality rates decreased after the core pandemic years, excess mortality remained higher than expected based on prepandemic levels. The largest portion of 2023 excess mortality was driven by drug poisoning, but many other external and natural causes exceeded what prior trends would have projected.

Increases in early adult mortality can signal population risks that may become more pronounced as these cohorts age. These results suggest the possibility of a worsening mortality crisis unless these trends are reversed. Policy solutions will require attention to the underlying causes of intensifying excess mortality among early adults (eg, opioid use, alcohol consumption, traffic safety, dietary risks). The 2 distinct phases of increasing mortality (before and after 2020) may also suggest the need to attend to ongoing consequences of the COVID-19 pandemic—which may be expressed in causes of death related to long-term consequences of infection, medical disruption, and social dislocation—and to deleterious health trends that predated it. Limitations of this study include the use of provisional mortality data for 2023 and examination of aggregate trends rather than distinct subpopulations defined by race, ethnicity, and gender.

## Notes

Supplement 1.
eMethods.

Supplement 2.
Data Sharing Statement
