# Supplementary material for: Mortality Trends Among Early Adults in the United States, 1999-2023
Source: JAMA Netw Open. 2025 Jan 31;8(1):e2457538. doi: 10.1001/jamanetworkopen.2024.57538 (PMC11786229; doi:10.1001/jamanetworkopen.2024.57538)
Supplement: Supplement 1. — eMethods. [file jamanetwopen-e2457538-s001.pdf]

Supplemental Online Content

Wrigley-Field E, Raquib RV, Berry KM, Morris KJ, Stokes AC. Mortality trends among early adults in the United States, 1999-2023. *JAMA Netw Open*. 2025;8(1):e2457538. doi:10.1001/jamanetworkopen.2024.57538

eMethods.

This supplemental material has been provided by the authors to give readers additional information about their work.

© 2025 Wrigley-Field E et al. *JAMA Network Open*.

eMethods

Data Sources

Cause-specific death counts: CDC WONDER Current Final Multiple Cause of Death Data 1999 - 2020 for the years 2000-2019 and the Provisional Multiple Cause of Death Data 2018 - Last Week for the years 2020-2023.

Mid-year population estimates: Census Bureau 1990 base for 1999, 2000 base for 2000-2009, 2010 base for 2010-2019, 2020 base for 2020-2023

Rationale for Querying Approach

Estimates of monthly cause-specific mortality rates were calculated using cause-specific mortality counts extracted from CDC WONDER. We selected the age group 25-44. Each cause of death was extracted separately, using the underlying cause of death definition and specific ICD-10 codes detailed in **Table S1**.

Underlying causes of death were selected in order to maintain mutually exclusive and exhaustive categories among causes of death. In some cases, use of this definition may underestimate the contribution of causes of death to overall and excess mortality levels. For example, during the COVID-19 pandemic, approximately 87% of COVID-19 deaths appeared in the underlying cause of death field, with the remainder appearing elsewhere on the death certificate.[1]

Causes of death

Our cause of death categories are a simplified version of a schema used in a prior report on working-age mortality by the National Academies of Sciences, Engineering, and Medicine (NASEM).[2] Table S2 reports the ICD codes corresponding to the NASEM categories and our simplified categories. Our schema uses the 11 most deadly causes in 2023, with three changes: we add the relatively small hypertensive disease to the much larger category of ischemic heart disease to create a broader circulatory category; we group all cancers together; and we retain respiratory causes as a distinct category.

Population denominators

Monthly population denominators were interpolated from the mid-year (July 1st) population estimates from the US Census Bureau, for each single year age and then summed to the age-group 25-44.

Observed death rates

Monthly cause-specific rates per 100,000 person-years were calculated by dividing the monthly death counts with the monthly population denominators and multiplying by 100,000.

© 2025 Wrigley-Field E et al. *JAMA Network Open*.

Expected death rates

First-differenced seasonal ARIMA models were fit to the crude monthly observed mortality rates for each cause of death for 1999-2010 and then projected forward for 2011-2023. The auto.arima() function under the forecast package was implemented in R to fit the ARIMA models. The function fits multiple models based on the input constraints and compares Akaike Information Criterion (AIC) values. The best model was selected by minimizing the AIC value. The maximum seasonal auto-regression term was set to 1 to limit the seasonal dependencies to the most recent seasonal lag. The standard deviation of the residuals between the fitted values and the observed values for each cause of death ranged from 0.08 to 0.28, indicating good model fit. To estimate the projected rates with corresponding confidence intervals, we used a bootstrapping method to create 10,000 simulated projections and took the mean and distribution, as described elsewhere.[3]

The years 1999-2010 were used as the baseline—consistent with other work[2,4]—because 2010 was identified as having the lowest observed all-cause mortality rate for the 25-44 age group within the 1999-2023 period. As Figure 1 makes clear, not all causes of death began to increase in the same year; when a cause of death remained on its pre-2011 trajectory into the 2010s (e.g., digestive causes and homicides), it simply did not have excess mortality until the year that it deviated from that trajectory. The flexibility of the cause-specific analyses allows us to identify deviations from the pre-2011 trends when they exist without assuming that they do, thereby capturing these variations across causes.

Expected and excess deaths

Monthly projected death rates were multiplied by monthly population estimates and summed by year to estimate expected death counts. Expected deaths were subtracted from observed deaths to estimate excess death counts. Excess death rates were estimated as the difference between observed and projected death rates.

Evaluation of the sensitivity of the results to age standardization

We examined the sensitivity of the results to age standardization within the 25-44 age category. We performed age standardization using the 2000 mid-year population distribution for the 10-year age groups 25-34 and 35-44. The difference between the all-cause excess death estimates for the crude and age standardized analyses was less than 2%. Therefore, in the main text, we only report the results from the crude analysis.

Further information available

Raw data, code, and visual representations of observed death rates and ARIMA projections for each cause of death are available at https://osf.io/j47kq/.

© 2025 Wrigley-Field E et al. *JAMA Network Open*.

Table S1. Mortality Data Queries

Multiple Cause of Death, 1999-2020 Request			
**1. Organize table layout:**	**3. Select demographics:**	**4. Select time period of death:**	**6. Select underlying cause of death:**
**Group Results By**	**Ten-Year Age Groups**	**Year**	**Advanced Finder Options**
Month	25-34 years<br>35-44 years	1999<br>2000<br>2001<br>2002<br>2003<br>2004<br>2005<br>2006<br>2007<br>2008<br>2009<br>2010<br>2011<br>2012<br>2013<br>2014<br>2015<br>2016<br>2017<br>2018<br>2019	Select ICD-10 codes for each specific cause of death separately (see Table S2)
**Provisional Mortality Statistics, 2018 through Last Week Request**			
**1. Organize table layout:**	**3. Select demographics:**	**4. Select time period of death:**	**6. Select underlying cause of death:**
**Group Results By**	**Ten-Year Age Groups**	**Year**	**Advanced Finder Options**
Month	25-34 years<br>35-44 years	2020<br>2021<br>2022<br>2023*<br>(*provisional)	Select ICD-10 codes for each specific cause of death separately (see Table S2)

© 2025 Wrigley-Field E et al. *JAMA Network Open*.

Table S2. Cause of Death Categories and Associated ICD-10 Codes

**NASEM Causes**	**ICD10 Codes**	**Simplified Causes**
Alcohol-induced	E24.4, G31.2, G62.1, G72.1, I42.6, K70, K29.2, K85.2, K86.0, R78.0, X45, X65, Y15, F10	Alcohol-related
Non-HIV/AIDS infectious and parasitic	A00–A99, B00–B19, B25–B99	Other natural
Diseases of the nervous system	G00–G98 (excluding G31.2, G62.1, G72.1)	Other natural
Diseases of the respiratory system	J00–J98	Respiratory
Diseases of the digestive system	K00–K92 (excluding K29.2, K85.2, K86.0)	Digestive
Diseases of the genitourinary system	N00–N98	Other natural
Other external	W00–W99, X00–X39, X46–X59, Y16–Y36, Y40–Y84, Y86, Y87.2, Y88, Y89	Other external
All other causes	D50–D89, H00–H57, H60–H93, L00–L98, M00–M99, O00–O99, P00–P96, Q00–Q99, R00–R99 (excluding R78.0), U00–U99	Other natural
Liver cancer	C22	Cancer
Lung cancer	C33, C34	
All other cancers	C00–D49 (excluding C22, C33, C34)	
Drug poisoning	X40–X44, X60–X64, X85, Y10–Y14<br>F11–F16, F19	Drug poisoning
Endocrine, nutritional and metabolic diseases	E00–E88 (excluding E24.4)	Endocrine, nutritional and metabolic diseases
HIV/AIDS	B20–B24	Other natural
Homicide	X86–X99, Y00–Y09, Y87.1	Homicide
Hypertensive disease	I10–I15	Circulatory
Ischemic heart disease and other diseases of the circulatory	I00–I99 (excluding I10–I15, I42.6)	Circulatory
Mental and behavioral (excluding drug poisoning and alcohol-related)	F01–F09, F17, F18, F20-F99	Other natural
Suicide	X66–X84, Y87.0	Suicide
Transport	V01–V99, Y85	Transport

© 2025 Wrigley-Field E et al. *JAMA Network Open*.

NASEM: National Academies of Sciences, Engineering, and Medicine

Supplemental References

1. National Center for Health Statistics. Guidance for Certifying Deaths Due to Coronavirus Disease 2019 (COVID–19): Expanded in February 2023 to Include Guidance for Certifying Deaths Due to Post-acute Sequelae of COVID-19. 2023. NCHS Vital Statistics Rapid Release Reports. 02/27/2023. https://stacks.cdc.gov/view/cdc/124588
2. National Academies of Sciences, Engineering, Medicine. High and Rising Mortality Rates Among Working-Age Adults. The National Academies Press; 2021:596.
3. Hyndman RJ, Athanasopoulos G. Forecasting: principles and practice. 3rd edition ed. OTexts; 2021.
4. Abrams LR, Myrskylä M, Mehta NK. The “double jeopardy” of midlife and old age mortality trends in the United States. Proceedings of the National Academy of Sciences. 2023-10-17 2023;120(42)doi:10.1073/pnas.2308360120

© 2025 Wrigley-Field E et al. *JAMA Network Open*.
