# Supplementary material for: Mortality Trends Among Early Adults in the United States, 1999-2023
Source: JAMA Netw Open. 2025 Jan 31;8(1):e2457538. doi: 10.1001/jamanetworkopen.2024.57538 (PMC11786229; doi:10.1001/jamanetworkopen.2024.57538)
Supplement: Supplement 2. — Data Sharing Statement [file jamanetwopen-e2457538-s002.pdf]

Data Sharing Statement

Wrigley-Field. Mortality Trends Among Early Adults in the United States, 1999-2023. *JAMA Netw Open*. Published January 31, 2025. doi:10.1001/jamanetworkopen.2024.57538

Data
Data available: Yes
Data types: Deidentified participant data
How to access data: All data and statistical code are posted at https://osf.io/j47kq/
When available: With publication

Supporting Documents
Document types: Statistical/analytic code
How to access documents: All data and statistical code are posted at https://osf.io/j47kq/
When available: With publication

Additional Information
Who can access the data: Anyone (open internet link)
Types of analyses: Any purpose
Mechanisms of data availability: Open internet (i.e., freely available)
